# The Impact of Deep Y Descent on Hemodynamics in Patients With Heart Failure and Preserved Left Ventricular Systolic Function

**DOI:** 10.3389/fcvm.2021.770923

**Published:** 2021-12-02

**Authors:** Daisuke Harada, Hidetsugu Asanoi, Takahisa Noto, Junya Takagawa

**Affiliations:** ^1^The Cardiology Division, Imizu Municipal Hospital, Toyama, Japan; ^2^Toyama Nishi General Hospital, Toyama, Japan

**Keywords:** heart failure, right ventricular diastolic function, atrial fibrillation, hemodynamics, left ventricular ejection fraction, deep Y descent, right heart catheterization, HFpEF - heart failure with preserved ejection fraction

## Abstract

**Background:** Influence of right ventricular diastolic function on the hemodynamics of heart failure (HF). We aimed to clarify the hemodynamic features of deep Y descent in the right atrial pressure waveform in patients with HF and preserved left ventricular systolic function.

**Methods:** In total, 114 consecutive inpatients with HF who had preserved left ventricular systolic function (left ventricular ejection fraction ≥ 50%) and right heart catheterization were retrospectively enrolled in this study. The patients were divided into two groups according to right atrial pressure waveform, and those with Y descent deeper than X descent in the right atrial pressure waveform were assigned to the deep Y descent group. We enrolled another seven patients (two men, five women; mean age, 87 ± 6) with HF and preserved ejection fraction, and implanted a pacemaker to validate the results of this study.

**Results:** The patients with deep Y descent had a higher rate of atrial fibrillation, higher right atrial pressure and mean pulmonary arterial pressure, and lower stroke volume and cardiac index than those with normal Y descent (76 vs. 7% *p* < 0.001, median 8 vs. 5 mmHg *p* = 0.001, median 24 vs. 21 mmHg *p* = 0.036, median 33 vs. 43 ml/m^2^ *p* < 0.001, median 2.2 vs. 2.7 L/m^2^, *p* < 0.001). Multiple linear regression revealed a negative correlation between stroke volume index and pulmonary vascular resistance index (wood unit*m^2^) only in the patients with deep Y descent (estimated regression coefficient: −1.281, *p* = 0.022). A positive correlation was also observed between cardiac index and heart rate in this group (r = 0.321, *p* = 0.038). In the other seven patients, increasing the heart rate (from median 60 to 80/min, *p* = 0.001) significantly reduced the level of BNP (from median 419 to 335 pg/ml, *p* = 0.005).

**Conclusions:** The hemodynamics of patients with HF with deep Y descent and preserved left ventricular systolic function resembled right ventricular restrictive physiology. Optimizing the heart rate may improve hemodynamics in these patients.

## Introduction

Right ventricular (RV) function affects the severity of heart failure independently of left ventricular (LV) systolic function (1). The right ventricle is characterized by higher compliance than the left ventricle, and the diastolic function of the right ventricle is superior to that of the left ventricle (2, 3). Therefore, RV diastolic function plays an important role in hemodynamics, and the function should be examined to consider the therapeutic strategies for patients with heart failure. The deceleration time and velocity of tricuspid inflow available from echocardiography are applied to the evaluation of RV diastolic function; however, its clinical significance is not confirmed and the assessment of the function may be difficult because of the anatomy of the right ventricle (4, 5). The waveform of the jugular venous pulse has been used to infer the RV diastolic function (6). X descent is deeper than Y descent and is the lowest point of the jugular venous pulse, which indicates that RV diastolic function is normal. On the other hand, the Y descent becomes rapid and deeper than the X descent, which is derived from two patterns of the RV diastolic pressure-volume curve. One is the right-sided shift of the RV diastolic pressure-volume curve and volume overload, and the representative disease is tricuspid regurgitation. The other is an upward shift of the RV diastolic pressure-volume curve and restriction filling, and representative diseases are restrictive cardiomyopathy and constrictive pericarditis (3, 6, 7). These changes reduce the reserve function of RV distensibility or less-distensible right ventricle. Thus, Y descent deeper than X descent indicates an abnormal pattern in the jugular venous pulse or right atrial pressure waveform and suggests a less-distensible right ventricle. We demonstrated that more common features, such as aging, atrial fibrillation (AF), and increase in RV systolic pressure, are associated with the sign in patients with preserved systolic function, and that the feature is, by no means, unique to specific diseases, such as tricuspid regurgitation, constrictive pericarditis, and restrictive cardiomyopathy (8). Y descent deeper than X descent may be an essential therapeutic target of heart failure; however, little attention has been paid to the hemodynamics of the sign in patients with heart failure and preserved LV systolic function. By right heart catheterization (RHC), we investigated the impact of deep Y descent on the hemodynamics in patients with deep Y descent in the right atrial pressure waveform and preserved systolic function in this study.

## Methods

### Study Population

**Figure 1:**
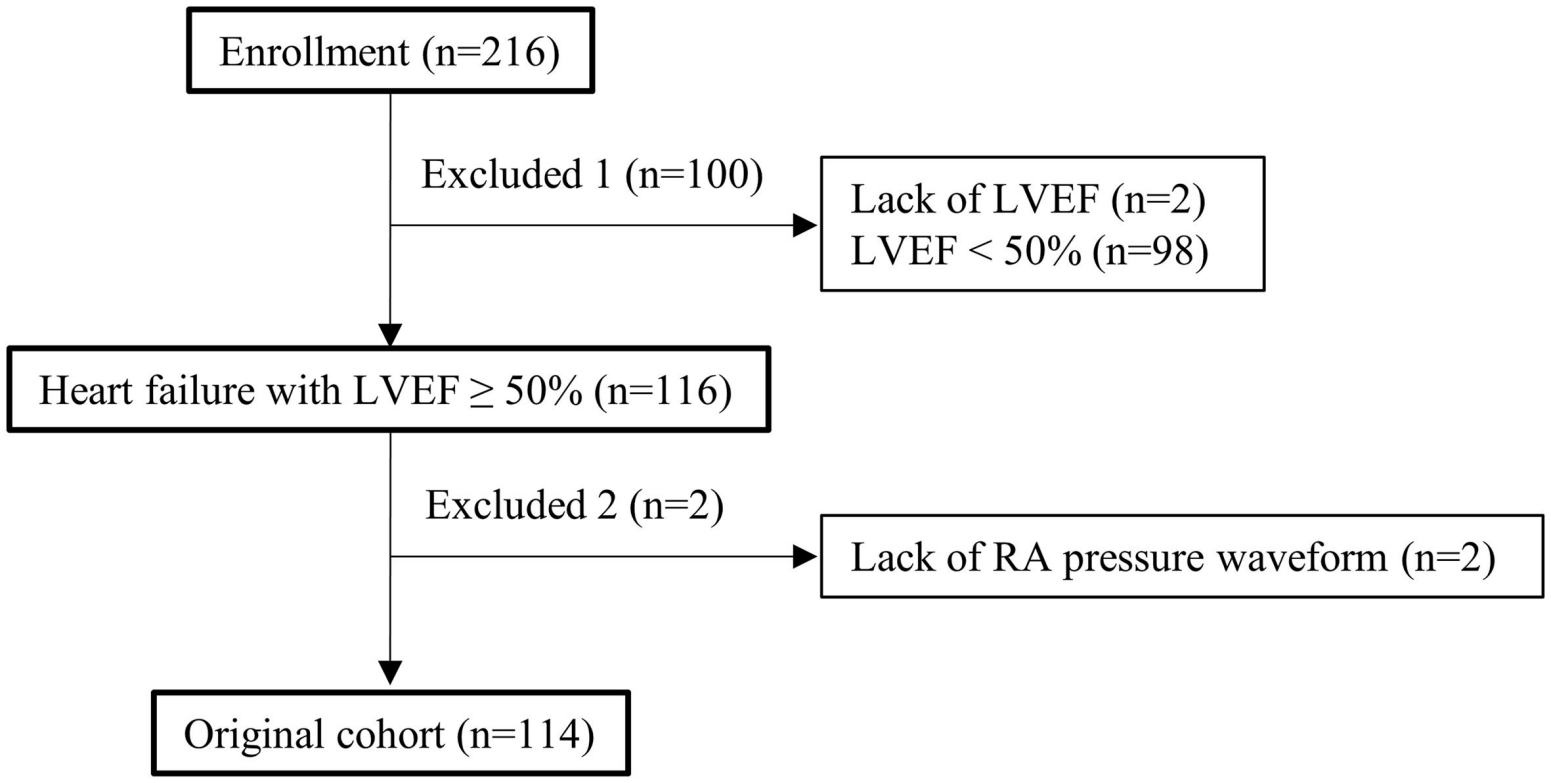
Study flowchart. LVEF, left ventricular ejection fraction; RA, right atrial.

In this study, after receiving approval from the Human Subject Review Committee of our institute, all data from the RHC database and medical records were retrospectively obtained. Between April 2011 and March 2020, 216 consecutive inpatients with heart failure who underwent RHC to assess their hemodynamics were enrolled (Figure 1). Patients were excluded if they had LV ejection fraction (LVEF) <50% and lacked data such as LVEF and right atrial waveform available from RHC. In total, 114 patients were enrolled in this study. Echocardiography and blood testing of all the patients were performed within 5 days before and after RHC. The patients were divided into two groups according to the right atrial pressure waveform. We defined LVEF ≥ 50% as preserved LV systolic function. To validate clinical application based on this study, seven more patients were enrolled in an additional study. The details are discussed below. Based on the arrangement of our hospital, informed consent was provided by all the patients. The study complied with the Declaration of Helsinki.

### Assessment of Echocardiography

Echocardiography with Vivid 7 (General Electric Healthcare, Wauwatosa, WI, United States) was performed using standard methods (5, 9, 10). LV end-diastolic and end-systolic volumes were measured using modified Simpson's method. The LVEF was calculated as stroke volume divided by end-diastolic volume. We measured early diastolic velocities (e') using pulsed-wave tissue Doppler from the apical view. The left atrial volume index was obtained using the biplane method from both the apical four- and two-chamber views. In addition, a tricuspid regurgitant jet was detected using the continuous Doppler technique to measure the systolic pulmonary arterial pressure (SPAP) (5).

### Assessment of Hemodynamics by Right Heart Catheterization

Right heart catheterization (RHC) was performed by experienced cardiologists using a standard method (11). Electrocardiography and sphygmomanometer were applied to non-invasively measure the heart rate and systemic blood pressure during the procedure. The measurement of cardiac output was conducted with the thermal dilution technique, but if the patient had tricuspid regurgitation and/or congenital heart disease, we used the Fick method, which is an alternative to the thermal dilution technique. For patients with atrial septal defects, we referred to the methods reported in a technical book (12). Cardiac index (CI) was calculated as the cardiac output divided by body surface area. The other parameters measured by RHC were as follows: right atrial pressure, RV systolic and end-diastolic pressure, RV positive and negative dp/dt, pulmonary arterial pressure, and pulmonary capillary wedge pressure. Pulmonary vascular resistance index (PVRI) was calculated as (mean pulmonary arterial pressure minus pulmonary capillary wedge pressure) divided by CI. Systemic vascular resistance index (SVRI) was calculated as (mean systemic blood pressure minus right atrial pressure) divided by CI. In this study, patients with X descent deeper than Y descent in the right atrial pressure waveform were assigned to the normal Y descent group; on the other hand, patients with Y descent deeper than X descent in the right atrial pressure waveform were assigned to the deep Y descent group.

### Effects of Heart Rate Modification on Patients With Heart Failure With Preserved Ejection Fraction (Additional Study)

**Figure 2:**
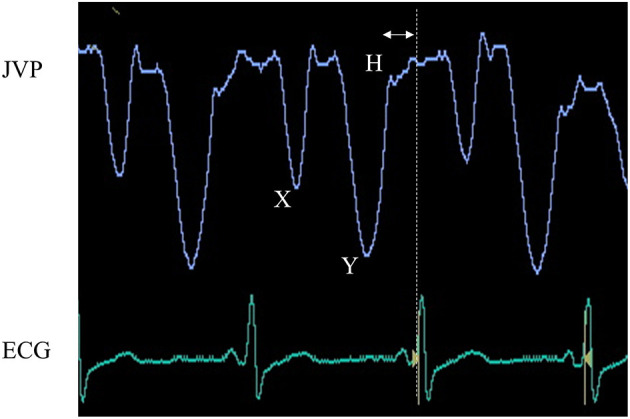
Juglar venous pulse waveform and electrocardiography. The jugular venous pulse waveform was recorded from an 87-year-old woman with heart failure with preserved ejection fraction and sinus arrest. This waveform shows a dominant “Y” descent deeper than that of “X” and is accompanied by an “H” wave, highly indicative of decreased right ventricular distensibility. To determine optimal heart rate, we shortened the diastolic period by the time between the end of the Y descent and the next R wave on electrocardiography (double-headed arrow).

The phases of diastole are isovolumic relaxation and filling. The filling phase is divided into the early rapid filling, slow filling period, and atrial contraction. In the filling phases, heart rate influences mainly the duration of the slow filling period and atrial contraction (13). In restrictive ventricular physiology, ventricular filling depends exclusively on the rapid filling phase, whereas the slow filling period and atrial contraction phase contribute little to the filling (14). Therefore, preventing bradycardia is recommended (15); however, under these conditions, shortening the slow filling period by increasing heart rate does not limit ventricular filling within a cardiac cycle, but may instead increase cardiac output in a rate-dependent manner. To assess the effectiveness of heart rate modification, we enrolled another seven patients with heart failure and preserved ejection fraction (HFpEF) and deep Y descent, and implanted a pacemaker. In this study, we defined patients with HFpEF as those with LVE F ≥ 50%, ≥ 2 positive variables of LV diastolic dysfunction, having symptoms and/or signs of heart failure, and a brain natriuretic peptide (BNP) level > 35 pg/ml (16, 17). Representative jugular venous pulse waveform in patients with HFpEF and deep Y descent is shown in Figure 2. We calculated the R-R interval and the interval from the end of Y descent to the next R wave on electrocardiography (Figure 2, double-headed arrow). We estimated optimal heart rate following the formula;

(Optimal R-R interval) = (R-R interval)–(the interval from the end of Y descent to the next R wave on electrocardiography).

To validate the results of this study, we examined whether sustained increases in heart rate for 3 months improved clinical indices.

### Statistical Analysis

Numerical data are expressed as the median (interquartile range) or mean ± SD. Student's *t*-test or Mann–Whitney U test was performed to compare parametric data between the groups. The chi-square test or Fisher's exact test was performed to compare non-parametric data between the groups. Repeated measures ANOVA or the Friedman test was performed to compare the change in three measurements taken from the same individual. The relationship between X and Y was calculated using univariate linear regression. Parameters with a *p*-value <0.1 in the univariate linear regression were included in the multivariate analysis. Statistical analysis variance inflation factor was used to check for multicollinearity. Significance was established at *p* < 0.05. All the statistical analyses were carried out using EZR (Saitama Medical Center, Jichi Medical University, Saitama, Japan) (18).

## Results

### Patient Characteristics

Patient characteristics are shown in Table 1. Compared with patients with normal Y descent and preserved LV systolic function, patients with deep Y descent and preserved LV systolic function had a significantly higher rate of pericardial disease, AF, usage of digitalis and loop diuretics, larger left atrial volume index and RV mid-cavity, and a lower rate of usage renin-angiotensin system inhibitors and body mass index. BNP was also significantly higher in patients with deep Y descent and preserved LV systolic function than in those with normal Y descent and preserved LV systolic function

**Table 1:** Patient characteristics according to right atrial pressure waveform.

	**Normal Y descent and** **preserved LV systolic function** **(*n =* 72)**	**Deep Y descent and** **preserved LV systolic function** **(*n =* 42)**	***p*-value**
Age, years old	72 (62–80)	76 (69–82)	0.180
Male/Female	30/42	19/23	0.710
BMI, kg/m^2^	24.3 (21.7–26.7)	23.0 (19.6–25.1)	0.041
Hemoglobin, g/dl	12.5 ± 2.2	12.3 ± 2.1	0.620
Creatinine, mg/dl	0.76 (0.63–0.98)	0.81 (0.68–1.1)	0.234
Brain natriuretic peptide, pg/ml	179 (68–298) (*n =* 61)	258 (172–489) (*n =* 41)	0.017
Lifestyle disease			
Hypertension	55 (76)	25 (60)	0.058
Diabetes mellitus	15 (21)	4 (10)	0.192
Hyperlipidemia	22 (31)	16 (38)	0.419
Cardiac disease			
Hypertensive heart disease	29 (40)	17 (40)	1
Ischemic heart disease	18 (25)	8 (19)	0.465
Valvular heart disease	33 (46)	17 (40)	0.578
Hypertrophic cardiomyopathy	1 (1)	0 (0)	1
Congenital heart disease	4 (6)	1 (2)	0.650
Pulmonary hypertension	4 (6)	2 (5)	1
Pericardial disease	2 (3)	6 (14)	0.050
Cardiac amyloidosis	1 (1)	0 (0)	1
Atrial fibrillation	5 (7)	32 (76)	<0.001
Pacemaker	0 (0)	2 (5)	0.134
Medication			
ACEI/ARB	52 (72)	20 (48)	0.009
Beta-blockers	28 (39)	23 (55)	0.100
Ca antagonist	29 (40)	13 (31)	0.319
Isosorbide dinitrate	23 (32)	9 (21)	0.228
Digitalis	4 (6)	10 (24)	0.004
Loop diuretics	30 (42)	30 (71)	0.002
Aldosterone blockers	12 (17)	12 (29)	0.133
Echocardiography			
LAVI, ml/m^2^	43 (39–47)	48 (42–51)	0.001
LVEF, %	63 (59–72)	67 (61–70)	0.570
LVEDD, mm	50 ± 8	49 ± 7	0.476
RV mid-cavity, mm	25 (21–29) (*n =* 66)	30 (26–32) (*n =* 38)	<0.001

*Data are the number of patients (%), mean ± SD, or median (interquartile). ACEI, angiotensin-converting enzyme inhibitor; ARB, angiotensin-receptor blocker; BMI, body mass index; LAVI, left atrial volume index; LV, left ventricular; LVEDD, left ventricular end-diastolic dimension; LVEF, left ventricular ejection fraction; RV, right ventricular*.

### Hemodynamic Characteristics of Deep Y Descent

The results of hemodynamics assessed by RHC are shown in Table 2. Right atrial, RV systolic, pulmonary arterial, and pulmonary capillary wedge pressures were significantly higher in patients with deep Y descent and preserved LV systolic function than in those with normal Y descent and preserved LV systolic function. Patients with deep Y descent and preserved LV systolic function also had lower stroke volume index (SVI) and CI.

**Table 2:** The results of right heart catheterization.

	**Normal Y descent and** **preserved LV systolic function** **(*n =* 72)**	**Deep Y descent and** **preserved LV systolic function** **(*n =* 42)**	***p*-value**
Heart rate (/min)	64 (58–76)	75 (60–85)	0.065
Systolic BP (mmHg)	128 (116–144)	122 (105–137)	0.092
Diastolic BP (mmHg)	69 (57–76)	66 (60–74)	0.526
Mean BP (mmHg)	89 (77–101)	87 (76–93)	0.202
SVI (ml/m^2^)	43 (36–50)	33 (27–42)	<0.001
CI (L/min/m^2^)	2.7 (2.4–3.3)	2.2 (2.0–2.7)	<0.001
Right atrial pressure (mmHg)	5 (3–8)	8 (5–10)	0.001
RV systolic pressure (mmHg)	31 (24–39)	35 (30–41)	0.038
RV end-diastolic pressure (mmHg)	7 (5–11)	9 (5–10)	0.435
RV dp/dt	261 (198–395)	269 (195–375)	0911
RV -dp/dt	222 (274–165)	266 (324–201)	0.057
Systolic PA pressure (mmHg)	30 (24–39)	37 (30–43)	0.013
Diastolic PA pressure (mmHg)	13 (11–19)	16 (12–20)	0.069
Mean PA pressure (mmHg)	21 (16–27)	24 (19–29)	0.036
PCWP (mmHg)	12 (9–16)	15 (11–19)	0.011
PVRI (Wood units*m^2^)	2.8 (1.9–3.4)	3.6 (2.0–4.8)	0.097
SVRI (Wood units*m^2^)	31 (25–35)	33 (26–38)	0.091

*Data are the median (interquartile). BP, blood pressure; CI, cardiac index; LV, left ventricular; LVEF, left ventricular ejection fraction; PA, pulmonary arterial; PCWP, pulmonary capillary wedge pressure; PVRI, pulmonary vascular resistance index; RV, right ventricular; SVI, stroke volume index; SVRI, systemic vascular resistance index*.

### Linear Regression of Stroke Volume and Cardiac Index

**Figure 3:**
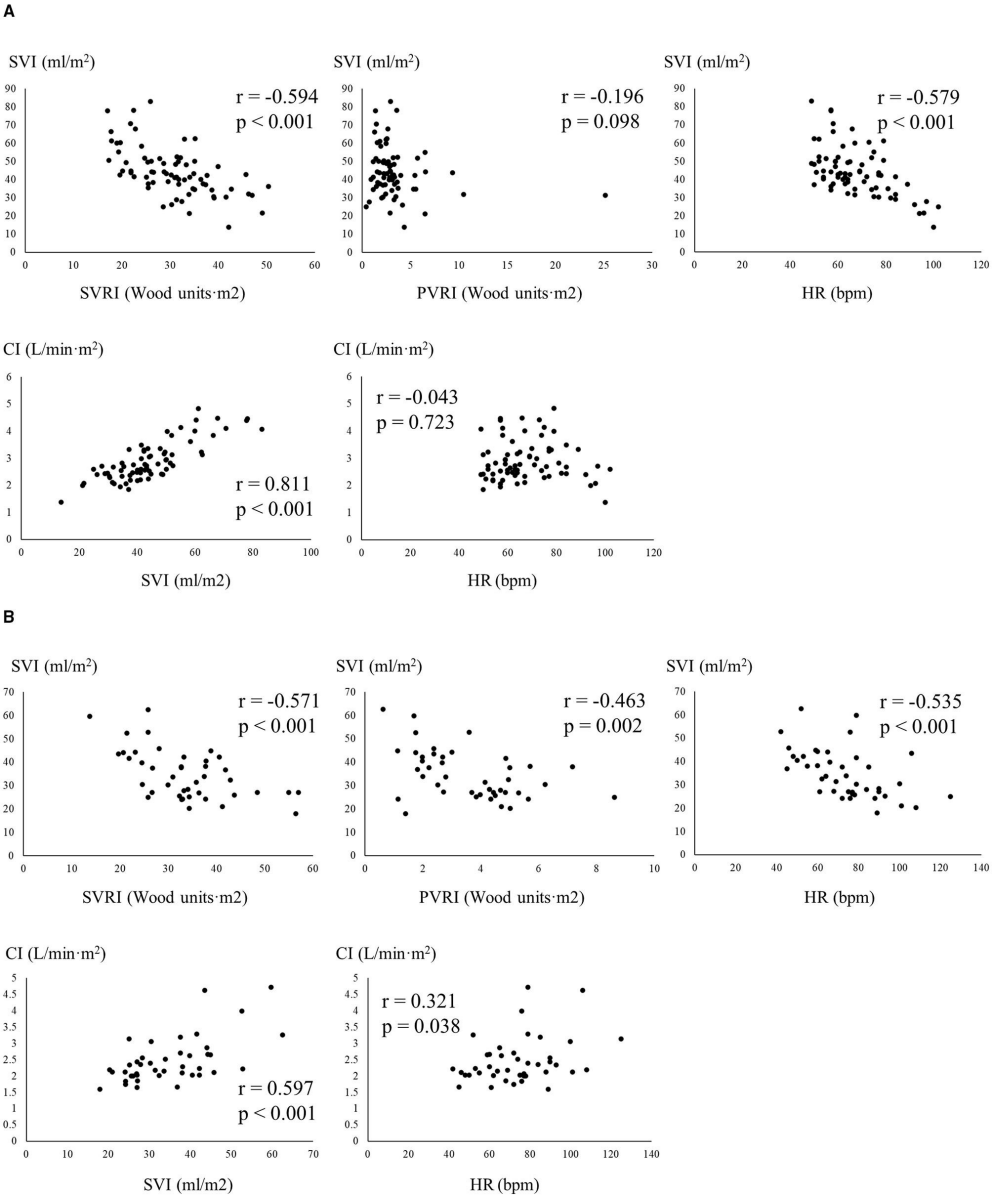
Relationship among indices of hemodynamics measured from right heart catheterization **(A)**, patients with normal Y descent and preserved LV systolic ejection fraction; **(B)**, patients with deep Y descent and preserved LV systolic function, respectively). CI, cardiac index; HR, heart rate; SVI, stroke volume index; SVRI, systemic vascular resistance index; PVRI, pulmonary vascular resistance index.

The results of single and multiple linear regression are shown in Figures 3A,B and Table 3. In multiple linear regression, the correlations between SVI and SVRI, and SVI and heart rate were negative in both groups; however, the negative correlation between SVI and PVRI was only observed in patients with deep Y descent and preserved LV systolic function. As shown in Figure 3B, a positive correlation was also observed between CI and heart rate in patients with deep Y descent and preserved LV systolic function.

**Table 3:** The relationship between SVI and other measurements available from right heart catheterization.

	**Normal Y descent**			**Deep Y descent**		
	**Univariate**	**Multivariate**		**Univariate**	**Multivariate**	
	***p*-value**	**Regression coefficient**	***p*-value**	***p*-value**	**Regression coefficient**	***p*-value**
Heart rate	<0.001	−0.539	<0.001	<0.001	−0.308	<0.001
Mean BP	0.171			0.172		
Right atrial pressure	0.602			0.644		
RV systolic pressure	0.319			0.349		
RV end-diastolic pressure	0.706			0.700		
RV dp/dt	0.031		0.090	0.602		
RV -dp/dt	0.156			0.389		
Mean PA pressure	0.649			0.060		0.800
PCWP	0.494			0.773		
PVRI	0.098		0.595	0.002	−1.281	0.022
SVRI	<0.001	−0.855	<0.001	<0.001	−0.707	<0.001

*Abbreviations are the same as in Table 2*.

### Effects of Heart Rate Modification

Clinical features in the other seven patients and the effectiveness of increasing the heart rate with a pacemaker on clinical characteristics are shown in Tables 4, 5, respectively. All the patients met the criterion for HFpEF and had deep Y descent in the jugular venous pulse waveform. Increasing the heart rate significantly reduced the level of BNP and was accompanied by improvement in echocardiographic measurements, such as mitral E/e' ratio and SPAP. Renal function was also improved by the alteration in heart rate.

**Table 4:** Clinical features in patients with HFpEF and deep Y descent on the day of heart rate modification.

	**Patients with HFpEF** **(*n =* 7)**
Age, years old	87 ± 6
Male/Female	2/5
BMI, kg/m^2^	21.5 ± 3.7
Underlying disease	
Hypertension	7 (100)
Diabetes mellitus	2 (29)
Hyperlipidemia	3 (43)
Prior coronary revascularization	2 (29)
Chronic obstructive pulmonary disease	1 (14)
Atrial fibrillation	4 (57)
Symptom and/or sign of heart failure	
Dyspnea on exertion	6 (86)
Leg edema	7 (100)
Neck Vein dilatation	7 (100)
Pleural effusion	2 (29)
Pacemaker indication and type of arrhythmia	
Chronic atrial fibrillation and bradycardia	4 (57)
Complete atrioventricular block	2 (29)
Sick sinus syndrome	1 (14)
Modes of cardiac pacing	
AAI	1 (14)
VVI	4 (57)
DDD	2 (29)
Medication	
ACEI/ARB	6 (86)
Beta-blockers	1 (14)
Ca antagonist	4 (57)
Isosorbide dinitrate	2 (29)
Loop diuretics	7 (100)
Aldosterone blockers	3 (43)

*Data are the number of patients (%), or mean ± SD. ACEI, angiotensin-converting enzyme inhibitor; ARB, angiotensin-receptor blocker; BMI, body mass index; HFpEF, heart failure with preserved ejection fraction*.

**Table 5:** Effects of heart rate modification in patients with HFpEF and pacemaker.

	**Patients with HFpEF, deep Y descent, and pacemaker (*n* = 7)**			
	**Before modification**	**On the day of modification**	**After 3 months of modification**	***p*-value**
Heart rate, bpm	60 (58–60)	60 (59–60)	80 (70–80)	0.001
Systolic BP, mmHg	114 (107–120)	107 (101–110)	106 (103–110)	0.276
Diastolic BP, mmHg	69 ± 14	66 ± 16	69 ± 15	0.564
Mean BP, mmHg	84 ± 13	80 ± 15	82 ± 14	0.766
Body weight, kg	48 ± 7	47 ± 6	45 ± 6	0.004
BUN, mg/dl	42 (24–65)	40 (23–66)	23 (22–30)	0.156
Cre, mg/dl	1.6 ± 0.5	1.7 ± 0.6	1.3 ± 0.6	0.007
BUN/Cre ratio	28 ± 13	27 ± 14	24 ± 9	0.026
eGFR, ml/min/1.73m^2^	27 ± 8	28 ± 10	36 ± 14	0.018
BNP, pg/ml	699 (425–744)	419 (368–753)	335 (280–405)	0.005
Echocardiography				
LAVI, ml/m^2^	42 (40–51)	44 (41–51)	48 (42–50)	0.341
LVEDD, mm	50 (48–52)	51 (48–52)	48 (47–51)	0.857
LVEF, %	63 ± 6	64 ± 7	63 ± 7	0.664
Mitral e', cm/s	5.4 ± 1.0	5.1 ± 0.8	5.2 ± 1.1	0.952
Mitral E/e ratio (septal)	21 ± 7	21 ± 7	17 ± 3	0.017
SPAP, mmHg	54 ± 8	53 ± 9	49 ± 10	<0.001
IVC, cm	23 ± 3	23 ± 3	21 ± 3	0.009

*Data are the mean ± standard deviation or median (interquartile range). BNP, brain natriuretic peptide; BP, blood pressure; BUN, blood urea nitrogen; Cre, creatinine; eGFR, estimated glomerular filtration rate; HFpEF, heart failure with preserved ejection fraction; IVC, inferior vena cava; LAVI, left atrial volume index; LVEDD, left ventricular end-diastolic dimension; LVEF, left ventricular ejection fraction; SPAP, systolic pulmonary arterial pressure*.

## Discussion

This study revealed that the resting hemodynamics of Y descent was deeper than that of X descent in the right atrial pressure waveform. Lower SVI and CI were observed in patients with deep Y descent. The hemodynamics of deep Y descent and preserved LV systolic function resembled RV restrictive physiology. Increasing the heart rate using a pacemaker improved the clinical condition of patients with HFpEF.

### Deep Y Descent and Hemodynamics in Patients With Heart Failure and Preserved Left Ventricular Systolic Function

Regarding the hemodynamic features of deep Y descent and preserved LV systolic function, a negative correlation was observed between SVI and PVRI, and right heart system pressures were high; a positive correlation was observed between CI and heart rate, and low CI was accompanied by low SVI. Unique hemodynamics was observed in patients with deep Y descent and preserved LV systolic function. The SVI in these patients significantly depended on PVRI, but this relationship was not observed in patients with normal Y descent. This suggested that RV preload reserve in patients with deep Y descent and preserved LV systolic function is limited by increased RV afterload or failure of the Frank-Staring mechanism (19). From the point of view of pressure-volume loop, the Frank-Starling mechanism failed because the RV diastolic pressure-volume curve in patients with deep Y descent and preserved LV systolic function shifted upward and/or to the right side compared with that in patients with normal Y descent. In general, increased ventricular preload occurs to compensate for the decrease in stroke volume due to ventricular systolic dysfunction and/or increased ventricular afterload, which leads to the ventricular diastolic pressure-volume curve shifting to the right side. Indeed, the mean pulmonary arterial pressure was higher and RV mid-cavity was larger in patients with deep Y descent and preserved LV systolic function. The Frank-Starling mechanism may have occurred, but SVI remained low compared with that in patients with normal Y descent. This suggested that RV dilatation was insufficient to compensate for the low stroke volume because of increasing RV afterload and the right ventricle losing high compliance. The right-sided shift of the RV diastolic pressure-volume curve was not enough to fulfill conditions of failure of Frank-Starling mechanisms in patients with deep Y descent and preserved LV systolic function. Ventricular diastolic dysfunctions, such as ventricular relaxation abnormality and impairment of ventricular distensibility, play a role in the upward shift of the ventricular diastolic pressure-volume curve (20), but RV negative dp/dt was the same between the groups in this study. Therefore, impairment of RV distensibility existed. Although the RV diastolic pressure-volume relationship was not examined directly, the shifts in the RV diastolic pressure-volume may have caused the failure of the Frank-Starling mechanism, which suggests that deep Y descent is indicative of a less-distensible right ventricle in patients with deep Y descent and preserved LV systolic function. In patients with deep Y descent, a positive correlation was observed between heart rate and CI, but the heart rate was also negatively correlated with SVI, and SVI was positively correlated with CI. These findings seemed to be contradictory to each other. Although cardiac output is in proportion to heart rate, resting cardiac output is kept constant to some extent even if the heart rate is increased because stroke volume is decreased by increasing heart rate (21, 22). This phenomenon arises from a decrease in end-diastolic volume due to an increase in heart rate or shortening the duration of the slow filling period and atrial contraction. However, in a less-distensible right ventricle, ventricular filling depends exclusively on the rapid filling phase, whereas the slow filling period and atrial contraction phase contribute little to the filling (14). Compared with normal Y descent, there was little influence of increasing heart rate on the SVI of patients with deep Y descent, which was represented in the difference of regression coefficient in Table 3. Thus, resting CI was increased rather than kept constant by increasing heart rate, and a positive correlation might be observed between resting cardiac index and heart rate in patients with deep Y descent.

### Clinical Implication

This study revealed that deep Y descent was a functional marker for RV diastolic function rather than a specific disease marker. Therefore, assessment of RV diastolic function, the cause of deep Y descent, and selection of therapeutic strategies suitable for less-distensible right ventricle are of importance in patients with heart failure and preserved LV systolic function, because the complication of less-distensible right ventricle deteriorates the hemodynamics.

#### Assessment of RV Diastolic Function

Nowadays, echocardiography is an important non-invasive tool for quantitative assessment of cardiac function, but in comparison with LV diastolic function, there is a lack of guidance for the assessment and quantification of RV diastolic function (23). Therefore, it is difficult to examine RV diastolic function. In this study, the waveform of right atrial pressure in RHC was applied to the assessment of RV diastolic function based on previous reports (6, 8). It is a qualitative evaluation, but the inference from pressure waveform may be needed to assess RV diastolic function.

#### The Cause of Deep Y Descent

As the essential hemodynamic feature of a less-distensible right ventricle was lower CI in this study, the cause of deep Y descent should be examined. Although constrictive pericarditis is the representative disease of the less-distensible right ventricle and preserved systolic function, the rate of AF was higher in patients with deep Y descent than in those with normal Y descent, which should be paid attention to. Previous studies have revealed that AF is associated with deep Y descent in the right atrial pressure or jugular venous pressure waveform (6, 8). Therefore, AF affects RV diastolic function. AF has been reported as an exacerbation factor for heart failure independently of LV systolic function (24); however, the influence on ventricular diastolic function has been mostly examined in LV diastolic function (25). As the mechanisms of impaired RV distensibility were unable to be examined in this study, the reasons why AF leads to less-distensible right ventricle are slightly more complicated, and several possible mechanisms are considered. To compensate for the reduction of stroke volume caused by atrial kick elimination, the Frank-Starling mechanism occurs, which leads to an increase in RV end-diastolic volume and shifting of the RV diastolic pressure-volume curve to the right side. AF with bradycardia may induce volume overload. Increasing the heart rate may also be a compensation mechanism for a decrease in CI; however, incomplete RV relaxation due to AF with tachycardia may shift the RV diastolic pressure-volume upward. AF itself may deteriorate RV diastolic function, similar to LV diastolic function (25). On the other hand, a less-distensible right ventricle leads to high right atrial pressure, which may make AF refractory through atrial remodeling. In any case, it is difficult to maintain CI because of a less-distensible right ventricle complicated by AF. We recently reported that rhythm control may improve RV diastolic function (26). Therefore, if possible, catheter ablation may be recommended before less-distensible right ventricle becomes irreversible. If the less-distensible right ventricle is refractory, therapeutic strategies suitable for RV restrictive physiology should be selected.

#### HFpEF and Less-Distensible Right Ventricle

Heart failure and preserved ejection fraction (HFpEF), representative heart failure with preserved LV systolic function, has multifaceted pathophysiology contributing to poor outcomes of the disease. Compared with heart failure with reduced LVEF, the therapeutic strategies of HFpEF have not been fully established yet, which remains an open issue in the world. We recently reported that less-distensible right ventricle is a clinical factor associated with phenotype, increase in posterior probabilities of developing cardiac events, and poorer outcomes of HFpEF (27–29). Therefore, RV diastolic function plays an important role in the hemodynamics of patients with HFpEF. To compensate for the low stroke volume due to increased RV afterload, volume overload usually occurs; however, the compensatory mechanism may be unsuccessful in patients with less-distensible right ventricle because RV preload is limited; thus, RV afterload mismatch is expected. Indeed, a low CI and high mean pulmonary arterial and right atrial pressures were observed in patients with deep Y descent and preserved LV systolic function in this study. Decreasing RV afterload may improve the hemodynamics of patients with less-distensible right ventricle and HFpEF, but the effectiveness of vasodilators on the pulmonary artery is unknown (30) because the cause of pulmonary hypertension is mainly pulmonary venous, not arterial hypertension, or both, in HFpEF. We have clarified previously that the complication of less-distensible right ventricle attenuates the effectiveness of beta-blockers on patients with HFpEF (31), and that maintaining the heart rate has been posited as a therapeutic strategy for patients with restrictive ventricular physiology because cardiac output depends on heart rate (15). Indeed, the positive relationship between CI and heart rate was only observed in patients with deep Y descent and preserved LV systolic function in this study. Heart rate modification may play an important role in improving hemodynamics in these patients. However, the usefulness of this concept for HFpEF and less-distensible right ventricle has not been fully established. Moreover, the optimal heart rate at rest remains unknown. Using the jugular venous pulse waveform, we set the resting heart rate using a pacemaker in order not to impair early diastolic inflow to the right ventricle. BUN/Cre ratio, a marker of “prerenal” renal dysfunction (32), was improved after modification of heart rate, which might indicate that cardiac output was increased. Increasing cardiac output moderated volume overload arising from the Frank-Starling mechanism to compensate for low cardiac output, as shown in Table 5 (decrease in body weight, inferior vena cava diameter, and septal E/e' ratio). Especially, a decrease in septal mitral E/e' ratio, a marker of LV filling pressure (33), might contribute to improvement in BNP level, because there is a strong correlation between the level of BNP and LV end-diastolic wall stress (34). Although a causal relationship among the clinical features was not fully clarified in this study, to the best of our knowledge, this is the first to demonstrate the hemodynamic significance of deep Y descent in patients with preserved systolic function, and that a higher heart rate *via* pacemaker can improve clinical features, such as BUN/Cre ratio, renal function, BNP, mitral E/e', and SPAP, in patients with HFpEF and less-distensible right ventricle. Therefore, to maintain an appropriate heart rate to support CI, a pacemaker may be needed, as in this study, in order to improve the poorer outcomes of patients with HFpEF and RV afterload mismatch.

### Study Limitation

Some limitations must be considered. This is a retrospective study conducted in a single center, and a retrospective study has limitations. Echocardiography and blood sample testing of all the patients were performed within 5 days before and after RHC but not simultaneously. RHC was performed on patients without discontinuation of their medicine. The heterogeneity of cardiac disease also influenced hemodynamics. Therefore, our results may have been affected. Our study was only performed at rest. Exercise and drug loading examinations were not performed. Marked hemodynamic change occurs during exercise; therefore, the right atrial pressure waveform may change because the assessment of RV distensibility judged by the right atrial pressure waveform is influenced by RV systolic function, relaxation ability, and RV preload and afterload (3). As LV pressure was not examined in this study, the impact of LV diastolic dysfunction on RV diastolic function, i.e., inter-ventricular interaction, is unclear. To assess RV distensibility, the right atrial pressure waveform, which is measured invasively by RHC, was needed; however, the jugular venous pulse waveform can be measured non-invasively, and it accurately reflects the right atrial pressure waveform (8). We validated the effectiveness of heart rate modification in patients with less-distensible right ventricle and HFpEF, but the cohort was small and the effect on clinical outcomes, such as cardiovascular events, was not clarified. Further clinical prospective studies are warranted to confirm our results.

## Conclusions

The hemodynamics of patients with heart failure with deep Y descent and preserved LV systolic function resembled RV restrictive physiology. Optimizing the heart rate may improve hemodynamics in these patients.

## Data Availability Statement

The raw data supporting the conclusions of this article will be made available by the authors, without undue reservation.

## Ethics Statement

The studies involving human participants were reviewed and approved by Imizu Municipal Hospital Ethics/Clinical Trial Review Committee. The patients/participants provided their written informed consent to participate in this study.

## Author Contributions

DH and HA worked on the conception, methodology, and formal analysis. Data collection were performed by DH, TN, and JT. DH wrote the manuscript. All authors approved the final version of the manuscript.

## Funding

HA declares that this study received funding from Sun Medical Technology Research Corp., Sumitomo Riko Company Limited., Century Medical, Inc., Teijin Pharma Limited., Nipro Corporation., and Medtronic Japan Co, Ltd. The funders were not involved in the study design, collection, analysis, interpretation of data, the writing of this article or the decision to submit it for publication.

## Conflict of Interest

HA declares that this study received funding from Sun Medical Technology Research Corp., Sumitomo Riko Company Limited., Century Medical, Inc., Teijin Pharma Limited., Nipro Corporation., and Medtronic Japan Co., Ltd. The funders were not involved in the study design, collection, analysis, interpretation of data, the writing of this article or the decision to submit it for publication. The remaining authors declare that the research was conducted in the absence of any commercial or financial relationships that could be construed as a potential conflict of interest.

## Publisher's Note

All claims expressed in this article are solely those of the authors and do not necessarily represent those of their affiliated organizations, or those of the publisher, the editors and the reviewers. Any product that may be evaluated in this article, or claim that may be made by its manufacturer, is not guaranteed or endorsed by the publisher.
